# Red blood cell distribution width combined with age as a predictor of acute ischemic stroke in stable COPD patients

**DOI:** 10.3389/fneur.2023.1165181

**Published:** 2023-06-05

**Authors:** Shikun Cai, Yao Li, Bo Sun, Kai Wang, Zongren Wan, Dan Yang, Xiangyang Tian, Liao Wu, Rong Zhu

**Affiliations:** ^1^Department of Neurology, The Affiliated Huaian No. 1 People's Hospital of Nanjing Medical University, Huaian, China; ^2^Department of Respiratory Medicine, The Huaian Clinical College of Xuzhou Medical University, Huaian, China; ^3^Department of Rheumatology and Immunology, The Affiliated Huaian No. 1 People's Hospital of Nanjing Medical University, Huaian, China

**Keywords:** acute ischemic stroke, chronic obstructive pulmonary disease, red blood cell distribution width, age, prediction

## Abstract

**Aim:**

This retrospective study aimed to investigate the independent clinical variables associated with the onset of acute cerebral ischemic stroke (AIS) in patients with stable chronic obstructive pulmonary disease (COPD).

**Method:**

A total of 244 patients with COPD who had not experienced a relapse within 6 months were included in this retrospective study. Of these, 94 patients hospitalized with AIS were enrolled in the study group, and the remaining 150 were enrolled in the control group. Clinical data and laboratory parameters were collected for both groups within 24 h after hospitalization, and the data of the two groups were statistically analyzed.

**Results:**

The levels of age, white blood cell (WBC), neutrophil (NEUT), glucose (GLU), prothrombin time (PT), albumin (ALB), and red blood cell distribution width (RDW) were different in the two groups (*P* < 0.01). Logistic regression analysis showed that age, WBC, RDW, PT, and GLU were independent risk factors for the occurrence of AIS in patients with stable COPD. Age and RDW were selected as new predictors, and the receiver operating characteristic curves (ROC) were plotted accordingly. The areas under the ROC curves of age, RDW, and age + RDW were 0.7122, 0.7184, and 0.7852, respectively. The sensitivity was 60.5, 59.6, and 70.2%, and the specificity was 72.4, 86.0, and 60.0%, respectively.

**Conclusion:**

The combination of RDW and age in patients with stable COPD might be a potential predictor for the onset of AIS.

## 1. Introduction

Stroke is caused by cerebral vascular occlusion or hemorrhage. Among all cerebral strokes, acute ischemic stroke (AIS) has the highest incidence (~87%). Currently, AIS is the leading cause of disability and the second most common cause of death worldwide. In the United States, a new stroke occurs every 40 s on average (1). Epidemiological studies indicate that the incidence of AIS is rapidly increasing, making it a major contributor to the global economic burden and a significant public health challenge (1–3).

AIS patients frequently have other systemic diseases, such as hypertension, diabetes mellitus, and chronic obstructive pulmonary disease (COPD), among others, previous studies have shown that these comorbidities may affect the morbidity, mortality, and long-term prognosis of AIS patients (4, 5), and COPD has also been identified as a possible risk factor for stroke (5).

Chronic obstructive pulmonary disease (COPD) is a chronic inflammation that affects the respiratory tract and lungs, causing restricted airflow and persistent respiratory system symptoms. Enhanced oxidative stress, systemic inflammation, and endothelial dysfunction play a crucial role in the pathophysiological process of COPD, even in the stable phase of the disease (6, 7). These processes in turn change blood flow resistance and promote thrombus formation. Recent studies have indicated that COPD is an independent risk factor for pulmonary embolism (PE) (8, 9). It is equally possible that these factors also participate in the pathophysiological process of cerebral infarction. This may be the underlying mechanism of why patients with COPD are more prone to suffer from stroke.

Recent research has shown that acute exacerbation of chronic obstructive pulmonary disease (AECOPD) increases the risk of myocardial infarction and cerebral ischemic stroke by sharply rising systemic inflammatory markers, such as fibrinogen and interleukin-6 (5). The elevated levels of these inflammatory markers might be related to the increased risk of thrombus formation and stroke events (10, 11). However, the situation may not be exactly the same in patients with COPD, whose clinical symptoms seem to be stable, and the risk of stroke is often overlooked in clinical practice. More attention should be paid to stable COPD patients who are prone to stroke. The purpose of this investigation is to assess the independent clinical variables associated with the onset of acute cerebral ischemic stroke in patients with stable COPD. Knowledge of these variables might provide rational clinical intervention for the prevention of AIS in patients with stable COPD.

## 2. Materials and methods

### 2.1. Study population

**Figure 1:**
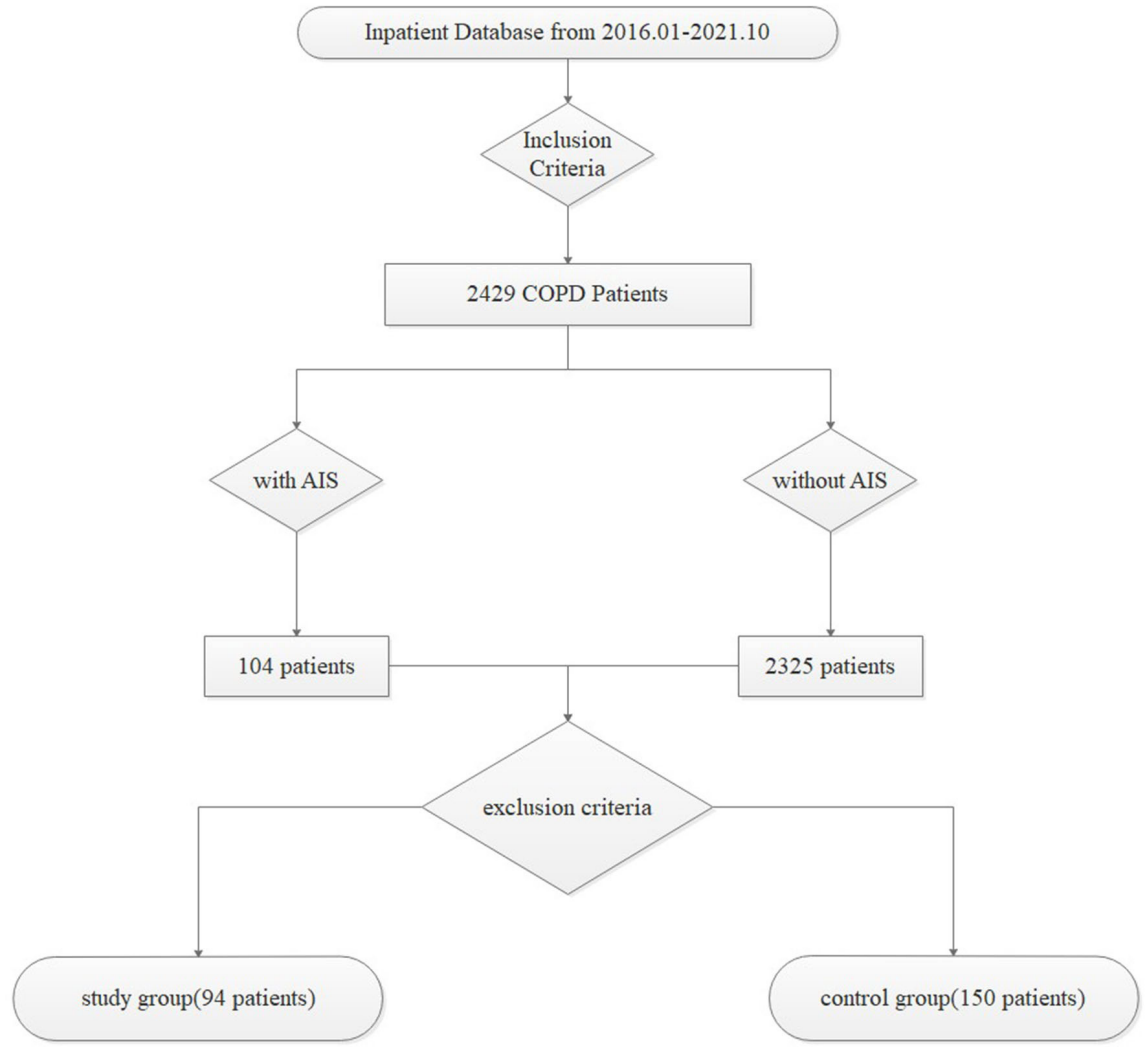
A flow diagram of the screening process.

A total of 244 patients with a prior diagnosis of COPD who had not experienced a relapse within 6 months were included in this retrospective study. Based on whether they had AIS, 94 patients hospitalized with AIS were enrolled in the study group, and the remaining 150 were enrolled in the control group. All data were collected from the patient database of Huai'an First People's Hospital from January 2016 to October 2021, and the screening process was demonstrated with a flow diagram in Figure 1.

The inclusion criteria were as follows: (1) patient age ≥ 40 years; (2) a post-bronchodilator FEV1/FVC ratio < 0.70, required for the diagnosis of COPD in all patients, along with a history of exposure to risk factors (such as smoke exposure) and/or with clinical symptoms (such as dyspnea, chronic cough, or sputum production) (12); (3) none of the patients had a relapse of COPD within 6 months; and (4) AIS was diagnosed by early diffusion-weighted magnetic resonance imaging (DWI-MRI) combined with symptoms of neurological impairment in the study group.

The exclusion criteria were as follows: (1) patients complicated with respiratory tract infection (such as AECOPD) or other systematic inflammatory diseases (such as rheumatic diseases, sepsis, pancreatitis, and shock); (2) patients with a negative bronchial dilation test; (3) patients complicated with hematological diseases (such as lymphoma, hemorrhagic diseases, hematologic malignancies, and anemia) or a recent medical history of bleeding or blood transfusion; (4) patients with renal insufficiency or hypohepatia; (5) patients with malignant tumors; (6) pregnant or lactating women; (7) patients with missing or incomplete data; and (8) patients with a previous history of AIS, exhibiting old cerebral infarction lesions in the MRI report, or taking anti-platelet drugs without a coronary atherosclerosis history, such as aspirin and clopidogrel.

The study was conducted in accordance with the Declaration of Helsinki and was approved by the ethics committee of Jiangsu Province Huai'an First People's Hospital (Ethics number: KY2022-011-01). Due to the retrospective nature of this study, informed consent was not obtained from all patients.

### 2.2. Data collection

We conducted a screening process to identify patients hospitalized with AIS, from which we selected a study group of 94 patients previously diagnosed with COPD who had not experienced a relapse within 6 months. In addition, we selected a control group of 150 stable COPD patients. We collected data on age, sex, body mass index (BMI), history including smoking history, basic diseases (including respiratory failure, hypertension, and diabetes), and laboratory tests (including routine blood tests, blood coagulation, and biochemical parameters) for both groups within 24 h of hospitalization.

Several previous studies have investigated stable COPD. For example, Hua et al. defined stable COPD as a clinically stable disease with no exacerbations in the last 30 days (13), while Coultas et al. (14) and Lin et al. (15) recruited outpatients as stable COPD patients. These studies provided a reference for our inclusion criteria to ensure that our patients were in a stable phase of COPD. According to the latest guidelines, a combination of neuroimaging in the clinically relevant area of the brain and symptoms of neurological impairment lasting more than 24 h is used to diagnose AIS (16, 17). Furthermore, the 2018 Guidelines for the Early Management of Patients with Acute Ischemic Stroke state that DWI-MRI is more sensitive than computed tomography (CT) for detecting AIS (17).

### 2.3. Statistical analysis

Quantitative data that displayed normal distribution and homogeneity of variance was expressed as (x ± S). To compare averages between the two groups, we used a two-independent sample *t*-test. When the data did not display normal distribution, we used the Mann–Whitney U-test. Qualitative data were expressed as percentages. To compare the data between groups, we used the Pearson chi-square (χ2) test, and when necessary, Fisher's exact probability test. Variables with a *p*-value < 0.1 in a one-way analysis of variables were considered independent variables in the logistic multifactor regression model. The occurrence of cerebral infarction was set as the dependent variable in stepwise logistic regression analysis. The *p*-values of independent variables included and removed during stepwise screening were set at 0.05. We drew the receiver operating characteristic (ROC) curve of each indicator to predict the occurrence of cerebral infarction. We estimated the 95% confidence interval (CI) of the area under the curve (AUC) using the Mann–Whitney method. We then compared the AUC value and selected the cutoff value when the Youden index reached its maximum. All tests were performed using a two-sided test, and a *p*-value of <0.05 was considered to be statistically significant. SPSS25.0 (SPSS Inc, Chicago, IL) was used for statistical analysis.

## 3. Results

### 3.1. Comparison of baseline characteristics

This retrospective study included a total of 244 patients who had previously been diagnosed with COPD and were currently in the stable phase. Of these, 94 patients who were hospitalized with AIS were assigned to the study group, while the remaining 150 patients comprised the control group. The results from Table 1 are as follows.

**Table 1:** Comparison of baseline demographic characteristics between the two groups.

**Baseline characteristics**	**COPD with AIS** **(*n* = 94)**	**COPD without AIS** **(*n* = 150)**	**T^a^ or Z^b^ or χ2^c^**	***p-*** **value**
Age (y) (mean ± SD)	78.56 ± 7.66	72.32 ± 7.44	6.159^a^	**< 0.001**
Gender (male) (*n*, %)	71 (75.53)	113 (75.33)	0.001^c^	0.972
BMI (kg/m^2^) (mean ± SD)	24.55 ± 3.45	23.78 ± 4.02	1.69^b^	0.091
Smoking History (N) (*n*, %)	76 (80.05)	96 (64)	7.889^c^	**0.005**
Respiratory failure History (N) (*n*, %)	85 (90.43)	145 (96.67)	4.162^c^	**0.041**
Hypertension (N) (*n*, %)	31 (32.98)	69 (46)	4.051^c^	**0.044**
Diabetes (N) (*n*, %)	71 (75.53)	132 (88)	6.426^c^	**0.011**

The bold values mean statistical significance.

a Student's *t*-test.

b Wilcoxon two-sample test.

c Chi-square test.

There was no significant difference in gender and average BMI between the study group and the control group. The proportion of male patients was 75.53% in the study group and 75.33% in the control group (χ^2^ = 0.001, *p* = 0.972), while the average BMI was 24.55 ± 3.45 in the study group and 23.78 ± 4.02 in the control group (Z = 1.69, *p* = 0.091). Similarly, there was no significant difference in smoking history, respiratory failure history, hypertension, and diabetes between the study and control groups. The smoking history was 80.05% in the study group and 64% in the control group (χ^2^ = 7.889, *p* = 0.005), the respiratory failure history was 90.43% in the study group and 96.67% in the control group (χ^2^ = 4.162, *p* = 0.041), hypertension was 32.98% in the study group and 46% in the control group (χ^2^ = 4.051, *p* = 0.044), and diabetes was 77.53% in the study group and 88% in the control group (χ^2^ = 6.426, *p* = 0.011). However, the age of patients in the study group was significantly higher than that of patients in the control group (78.56 ± 7.66 vs. 72.32 ± 7.44, *T* = 6.159, *p* < 0.001).

### 3.2. Comparison of laboratory parameters

Table 2 presents a comparison of all laboratory indicators. The results show that red blood cell (RBC), mean corpuscular volume (MCV), platelet (PLT), platelet distribution width (PDW), platelet crit (PCT), platelet-large cell rate (P-LCR), lymphocyte (LYM), eosinophil (EO), monocyte (MONO)%, EO%, thrombin time (TT), alanine aminotransferase (ALT), aspartate aminotransferase (AST), lactate dehydrogenase (LDH-L), triglyceride (TG), creatinine (CREA), and uric acid (UA) did not demonstrate any significant differences between the two groups.

**Table 2:** Comparison of the laboratory parameters between the two groups.

**Laboratory parameters**	**COPD with AIS(*n* = 94)**	**COPD without AIS (*n* = 150)**	***p-*** **value**
WBC (×10^9^/l)	7.91 ± 2.89	6.42 ± 1.64	**< 0.001**
RBC (×10^12^/l)	4.35 ± 0.61	4.50 ± 0.55	0.065
HB (g/l)	129.55 ± 19.13	137.18 ± 16.60	**0.002**
HCT (%)	39.66 ± 5.57	41.47 ± 5.46	**0.013**
MCV (fl)	91.32 ± 5.90	91.34 ± 12.04	0.134
MCH (Pg)	29.81 ± 2.21	30.91 ± 5.29	**0.031**
MCHC (g/l)	326.39 ± 12.62	329.88 ± 11.52	**0.027**
PLT (×10^9^/l)	207.18 ± 80.03	193.91 ± 67.40	0.263
RDW-SD (fl)	44.88 ± 3.93	43.05 ± 2.62	**< 0.001**
RDW-CV (%)	13.78 ± 1.24	12.92 ± 0.68	**< 0.001**
PDW (fl)	12.44 ± 3.43	13.29 ± 3.96	0.060
MPV (fl)	10.08 ± 1.70	10.64 ± 1.63	**0.019**
PCT (%)	0.21 ± 0.07	0.20 ± 0.06	0.721
P-LCR (%)	27.86 ± 9.78	30.61 ± 11.45	0.067
NETU (×10^9^/l)	5.78 ± 2.88	4.32 ± 1.69	**< 0.001**
LYM (×10^9^/l)	1.46 ± 0.65	1.53 ± 0.72	0.584
MONO (×10^9^/l)	0.53 ± 0.24	0.47 ± 0.27	**0.031**
EO (×10^9^/l)	0.15 ± 0.16	0.15 ± 0.16	0.664
NEUT (%)	70.23 ± 11.20	65.54 ± 10.45	**0.001**
LYM (%)	20.21 ± 9.29	24.45 ± 9.48	**0.003**
MONO (%)	7.02 ± 2.65	7.14 ± 2.12	0.469
EO (%)	2.14 ± 2.27	2.40 ± 2.42	0.287
PT (sec)	13.43 ± 1.14	12.87 ± 1.09	**< 0.001**
APTT (sec)	36.96 ± 10.44	34.29 ± 5.88	**0.030**
TT (sec)	18.17 ± 5.26	17.20 ± 2.19	0.580
FIB (g/l)	4.26 ± 2.47	3.42 ± 1.09	**0.001**
ALB (g/l)	37.86 ± 4.20	40.15 ± 4.50	**< 0.001**
ALT (μ/l)	15.67 ± 10.71	16.42 ± 9.50	0.103
AST (μ/l)	21.66 ± 9.19	20.31 ± 6.33	0.597
LDH-L (μ/l)	202.11 ± 71.77	182.41 ± 46.92	0.067
CHOL (mmol/l)	3.82 ± 1.01	4.12 ± 0.92	**0.003**
TG (mmol/l)	1.33 ± 1.02	1.34 ± 0.76	0.424
GLU (μmol/l)	6.29 ± 2.12	5.38 ± 1.51	**< 0.001**
CREA (μmol/l)	77.33 ± 25.37	70.60 ± 18.11	0.128
UA (μmol/l)	306.17 ± 113.23	325.17 ± 110.29	0.282

The bold values mean statistical significance.

However, the AIS in the stable COPD group displayed lower hemoglobin (HB, *p* = 0.002), hematocrit (HCT, *p* = 0.013), mean corpuscular hemoglobin (MCH, *p* = 0.031), mean corpuscular hemoglobin concentration (MCHC, *p* = 0.027), mean platelet volume (MPV, *p* = 0.019), MONO (*p* = 0.031), neutrophil (NEUT)% (*p* = 0.001), LYM% (*p* = 0.003), activated partial thromboplastin time (APTT, *p* = 0.030), fibrinogen (FIB, *p* = 0.001), and cholesterol (CHOL, *p* = 0.003) compared to the control group. White blood cell (WBC, 7.91 ± 2.89 vs. 6.42 ± 1.64, *p* < 0.001), NEUT (5.78 ± 2.88 vs. 4.32 ± 1.69, *p* < 0.001), glucose (GLU, 6.29 ± 2.12 vs. 5.38 ± 1.51, *p* < 0.001), and prothrombin time (PT, 13.43 ± 1.14 vs. 12.87 ± 1.09, *p* < 0.001) were significantly higher in the study group compared to the control group.

Furthermore, the levels of red blood cell distribution width standard deviation (RDW-SD) and red blood cell distribution width coefficient of variation (RDW-CV) in the study group were significantly increased compared to the control group (44.88 ± 3.93 vs. 43.05 ± 2.62, *p* < 0.001; 13.78 ± 1.24 vs. 12.92 ± 0.68, *p* < 0.001). Additionally, the albumin (ALB) of the study group decreased significantly compared to the control group (37.86 ± 4.20 vs. 40.15 ± 4.50, *p* < 0.001).

### 3.3. Logistic regression analysis

A total of 23 laboratory parameters with statistically significant differences were identified through the previous statistical analyses between the two groups. These parameters included smoking history, respiratory failure history, hypertension, diabetes, age, HB, HCT, MCH, MCHC, MPV, MONO, NEUT%, LYM%, APTT, FIB, CHOL, WBC, RDW-SD, RDW-CV, NETU, PT, ALB, and GLU. However, confounding factors were present. Therefore, we utilized the stepwise method in multiple logistic regression to screen for significant independent risk factors, which are summarized in Table 3. The identified risk factors were age, WBC, RDW-CV, PT, and GLU, with corresponding odds ratios of 1.074 (95% CI, 1.026–1.125; *p* = 0.002), 1.284 (95% CI, 1.082–1.523; *p* = 0.004), 2.268 (95% CI, 1.451–3.546; *p* < 0.001), 1.466 (95% CI, 1.045–2.055; *p* = 0.027), and 1.266 (95% CI, 1.044–1.535; *p* = 0.017), respectively, and are included in Table 4.

**Table 3:** Logistic regression model (stepwise regression analysis).

**Variable**	**Parameter**	**Parameter standard error**	**Wald statistics**	***p-*** **value**
Age	0.072	0.023	9.462	**0.002**
WBC	0.250	0.087	8.217	**0.004**
RDW-CV	0.819	0.228	12.91	**0.000**
PT	0.382	0.172	4.915	**0.027**
GLU	0.236	0.099	5.725	**0.017**

The bold values mean statistical significance.

**Table 4:** Independent risk factors of AIS (analyzed by logistic regression model).

**Variable**	**OR ratio**	**95% CI**	***P*** **-value**
Age	1.074	1.026–1.125	**0.002**
WBC	1.284	1.082–1.523	**0.004**
RDW-CV	2.268	1.451–3.546	**0.000**
PT	1.466	1.045–2.055	**0.027**
GLU	1.266	1.044–1.535	**0.017**

The bold values mean statistical significance.

### 3.4. Prediction values of RDW-CV combined with age in AIS

**Figure 2:**
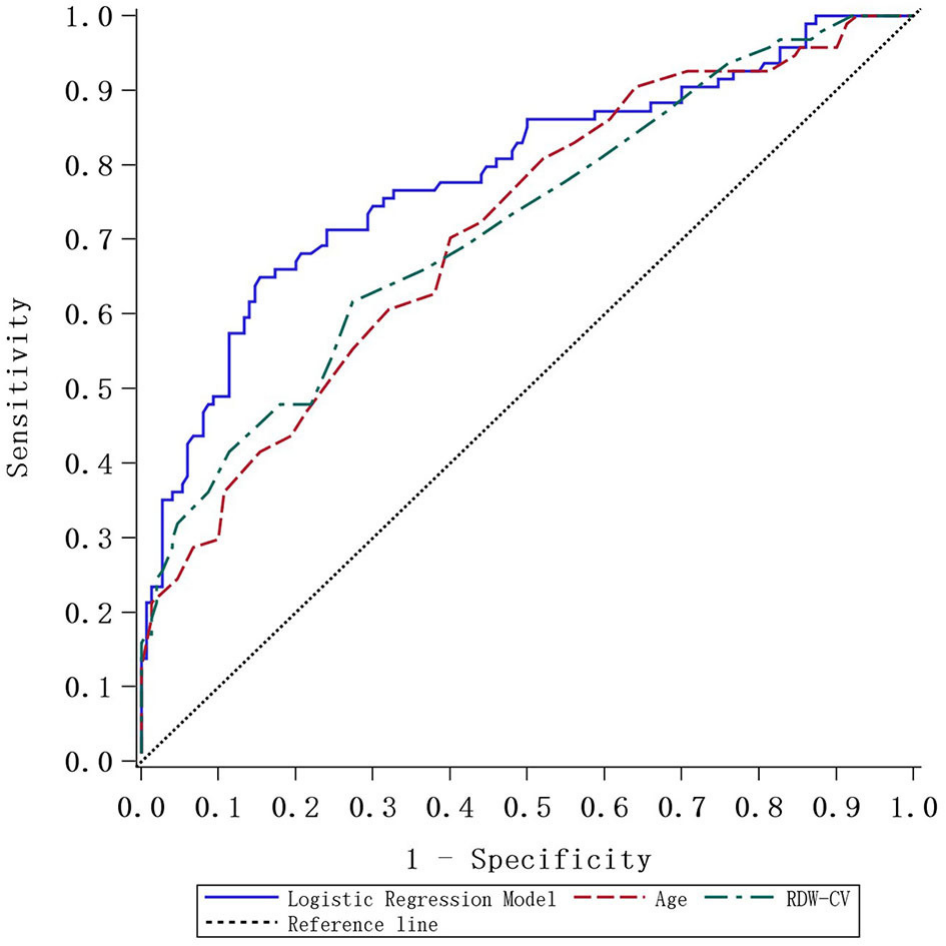
The ROC curve plotted according to the logistic regression model and comparative analysis.

Considering the long-term instability of WBC, PT, and GLU, age and RDW-CV were selected as new predictors. We also combined age with RDW-CV to create a new prediction model, and ROC curves were plotted accordingly. The detailed predictive accuracy values are illustrated in Table 5, and Figure 2 presents an intuitive comparison of ROC curves among these predictors. The AUC values for RDW-CV and age were 0.718 and 0.712, respectively, while the AUC for RDW-CV combined with age was 0.785. The optimal cutoff values of age combined with RDW-CV and RDW-CV ratio for predicting AIS were 74.5, 13.25, and 0.5, respectively, for age combined with RDW-CV and the RDW-CV ratio with corresponding sensitivities of 0.6, 0.724, and 0.86, respectively.

**Table 5:** Comparative analysis of the predictive value of age combined with RDW-CV, age, and RDW-CV.

**Parameters**	**AUC**	**95% CI**	**Cutoff value**	**Sensitivity (%)**	**Specificity (%)**
Age	0.7122	0.6461–0.7783	13.25	0.605	0.724
RDW-CV	0.7184	0.6520–0.7848	0.5	0.596	0.860
Age + RDW-CV	0.7852	0.7237–0.8467	74.5	0.702	0.6

## 4. Discussion

Medical research has demonstrated that there are multiple potential mechanisms linked to the pathogenesis of COPD, including systemic oxidative stress, inflammation, and others (18, 19). These conditions are also closely related to the development of cerebral disease (20). As a result, the risk of AIS in COPD patients, particularly in the stable phase, has captured our attention. In a previous study, Weimar et al. integrated age with the National Institution of Health stroke scale (NIHSS) score to assess the prognosis of stroke patients (21). Similarly, Turcato et al. incorporated age, NIHSS score, and other indices to predict early functional impairment after AIS (22). Wang et al. demonstrated that the RDW combined with D-Dimer could predict the occurrence of PE in patients with AECOPD (23). These preliminary results provide novel concepts for our investigation. To the best of our knowledge, we report for the first time potential relationships between age, RDW, and the occurrence of AIS in patients with stable COPD. Our findings suggest that older patients and those with elevated RDW are associated with the occurrence of AIS in patients with stable COPD.

The red blood cell distribution width is an index that reflects the variation of red blood cell (RBC) volume, which can be rapidly and conveniently accessed through a blood routine examination, and it is relatively stable in the normal population. However, it can change under certain physiological or pathological conditions. The most common use of RDW is for the diagnosis and evaluation of the treatment effect of anemia (24). In recent years, increasing studies have shown that RDW is closely related to the morbidity and prognosis of many diseases, such as myocardial infarction, heart failure, cerebrovascular diseases, and COPD (25–29). RDW can serve as a predictor of mortality in patients with COPD and it is also associated with poor clinical outcomes in AIS (27, 28). Furthermore, the value of RDW can predict the onset of AIS in patients with obstructive sleep apnea-hypopnea syndrome (29). However, the relationship between RDW and disease is not fully understood, and further research is necessary.

Current clinical studies have shown that inflammation and markers, such as tumor necrosis factor-alpha, interleukin-8, interleukin-10, and C-reactive protein (CRP), are known to be associated with RDW (30, 31). Systemic inflammatory cytokines have an adverse effect on the differentiation and maturation of RBCs (32). For instance, they can affect the stability of the red blood cell membrane, increase red blood cell fragility, inhibit red blood cell maturation, and reduce the blood cell longevity, leading to an increase in the variation in RBC size. All of these factors are significantly correlated with elevated RDW. In addition to inflammation, several studies have indicated that oxidative stress is also associated with RDW (33). Oxidative stress affects the longevity of RBCs and can disrupt the RBC membrane, increase the osmotic fragility of RBCs, and later the adhesion and aggregation ability of RBCs. These factors contribute to changes in the RDW index. Furthermore, these pathological processes promote coagulation and thrombus formation in cerebro-cardiovascular diseases (34, 35). In this study, we found that the level of RDW in the study group was higher than in the control group, which may be due to different levels of inflammation and oxidative stress between the two groups. Our logistic regression model analysis revealed that RDW was an independent risk factor for AIS in stable COPD patients. Therefore, inflammation and oxidative stress may be key factors linking increased RDW to the occurrence of AIS in patients with stable COPD. However, Shahsavarinia et al. concluded that RDW is not a significant predictive value for either stroke severity or stroke outcome in patients after tPA administration. They suggested that this may be due to the narrowing of the patient pool, and this negative result should be analyzed to understand the underlying mechanism (36).

Age was another independent risk factor for AIS in stable COPD patients in this study. Numerous studies have indicated that aging is an unchangeable factor for many diseases, and its potential influence mechanisms include promoting oxidative stress and inflammatory response (37–39). Age is relatively stable, but considering that RDW is influenced by some medical conditions, such as transfusion, anemia, malignancy, and others (40), we attempted to rule out as many influencing factors as possible. We used the stepwise method in multiple logistic regression to evaluate the contribution of risk factors in patients with stable COPD and regulate these influencing factors that may be confounders between the two groups. Although PT, WBC, and blood glucose were considered potential independent risk factors, these three factors were very easily influenced by conditions such as stress response. Therefore, due to the stability of the indicators, they were eliminated in the final result. After adjustment for these factors, increased RDW combined with age was clearly associated with the occurrence of AIS in patients with stable COPD. RDW and age are all relatively simple and readily available indices that can help doctors to better identify and assess high-risk patients with stable COPD and effectively prevent stroke as soon as possible.

There are several limitations to our research. First, the study was conducted at a single center, and the sample size was relatively small. Therefore, larger multicenter studies are required to validate our findings. Second, the retrospective nature of the study may attenuate its clinical significance. To address this issue, a well-designed prospective study is needed to confirm the predictive value of RDW and age for the onset of cerebral infarction in patients with stable COPD. Third, we did not stratify the patients into different subgroups based on pulmonary function tests, which may provide more accurate conclusions. Finally, although we considered inflammation as one of the potential reasons for cerebral infarction in stable COPD patients, we did not measure the levels of inflammatory factors in depth. This highlights the need for further investigation in this area. These limitations provide potential directions for future research.

In summary, this study demonstrates that the combination of age and RDW in patients with stable COPD may serve as a potential predictor for the onset of AIS. Although the underlying biological mechanisms remain uncertain, this approach provides a valuable and novel perspective for identifying the higher stroke risk in patients with stable COPD and may aid in disease prevention and progression.

## Data availability statement

The original contributions presented in the study are included in the article/Supplementary material, further inquiries can be directed to the corresponding author.

## Ethics statement

The studies involving human participants were reviewed and approved by Jiangsu Province Huai'an First People's Hospital. The ethics committee waived the requirement of written informed consent for participation.

## Author contributions

SC proposed the project idea, analyzed the data, and wrote the article. YL designed the experimental scheme and collected the data. ZW, DY, LW, and XT collected the data. KW and BS revised the article. RZ controlled the direction of the project. All authors contributed to the article and approved the submitted version.

## Supplementary material

SUPPLEMENTARY DATA SHEET 1 (1) In the gender, column 1 stands for male patients and 2 stands for female patients; (2) in the respiratory failure, hypertension, diabetes, and smoking history, columns 0 is for no and 1 is for yes; (3) in the group columns, 1 is for the study group and 2 is for the control group; (4) BMI is defined as a person's weight in kilograms divided by the square of the height in meters (kg/m^2^).
